# Cyclophosphamide use in treatment of refractory Kawasaki disease with coronary artery aneurysms

**DOI:** 10.1186/s12969-021-00526-0

**Published:** 2021-03-17

**Authors:** Olha Halyabar, Kevin G. Friedman, Robert P. Sundel, Annette L. Baker, Margaret H. Chang, Patrick W. Gould, Jane W. Newburger, Mary Beth F. Son

**Affiliations:** 1 Division of Immunology, Boston Children’s Hospital, 300 Longwood Avenue, Fegan 6, Boston, MA 02115 USA; 2 Department of Pediatrics, Boston Children’s Hospital, Harvard Medical School, Boston, MA 02115 USA; 3 Department of Cardiology, Boston Children’s Hospital, Boston, MA 02115 USA; 4 University of Pennsylvania Medical School, Philadelphia, PA USA

**Keywords:** Kawasaki disease, Coronary artery aneurisms, Treatment, Immunosuppression, Cyclophosphamide

## Abstract

**Background:**

Despite timely administration of IVIG, some patients with Kawasaki disease (KD) develop rapidly progressive or giant coronary artery aneurysms (CAA).

**Case presentation:**

We describe our experience using cyclophosphamide (CYC) for the treatment of such cases as well as a review of the literature on the use of CYC in KD. Through a retrospective chart review of our KD population, we identified ten children treated for KD with intravenous CYC (10 mg/kg/dose) for one or two doses. Seven patients were male, the median age was 2.0 years (range 4 months − 5 years). All patients received initial IVIG between day 4–10 of illness. Other anti-inflammatory treatments administered before CYC included second IVIG (*n* = 9), corticosteroids (*n* = 10), infliximab (*n* = 4), cyclosporine (*n* = 2), and anakinra (*n* = 1). Median illness day at administration of the first CYC dose was 22.5 days (range:10–36 days). The primary indication for treatment with CYC for all patients was large or giant CAA and/or rapid progression of CAA. Three patients received a second dose of CYC (10 mg/kg) for progressively enlarging CAA. CAA did not progress after final CYC treatment**.** One patient with a history of neutropenia in infancy developed severe neutropenia 9 days after treatment with CYC, which recovered without intervention or complications. No patient developed infections or other serious toxicity from CYC.

**Conclusion:**

In KD patients with severe and progressive enlargement of CAA despite anti-inflammatory therapy, CYC seemed to arrest further dilation and was well-tolerated. Future multicenter studies are needed to confirm our findings in this subgroup of KD patients.

## Background

Kawasaki disease (KD) is a childhood febrile illness and a medium vessel vasculitis primarily affecting the coronary arteries. Administration of high-dose intravenous immunoglobulin (IVIG) within 10 days of fever onset prevents coronary artery aneurysms (CAA) in most patients with KD. However, ~ 20% of KD patients develop CAA based on American Heart Association criteria with a z score ≥ 2.5 of the left anterior descending (LAD) and/or right coronary artery (RCA) in the first 6 weeks of illness. Approximately 1% of KD patients develop large or giant aneurysms (z score ≥ 10 or absolute coronary artery dimensions of ≥8 mm) [1, 2], a complication associated with significant long-term morbidity, and in rare cases, mortality [3, 4].

Treatments most commonly used to treat children with KD who fail to respond to initial therapy include additional doses of IVIG, corticosteroids, cytokine blockade including anti-TNF alpha and anti-Il-1 biologics, and cyclosporine [5–8].Cyclophosphamide (CYC) has been effective in the treatment of other severe pediatric vasculitides, and its use has been reported in the treatment of refractory KD [9–11].In this case series, we share our experience using CYC in the treatment of 10 patients with KD and large and/or rapidly progressive CAA.

## Methods

All KD patients treated between 2006 and 2019 were reviewed in our center’s database. Ten patients were treated with CYC and had complete data; their charts were extracted for clinical and imaging data elements. All echocardiograms from outside centers were uploaded and read by a single cardiologist (KF). CAA were defined according to the 2017 AHA Guidelines: z score of ≥2.5 of the LAD and/or RCA. Large or giant aneurysms defined by z score of ≥10 or absolute dimension of the CA ≥ 8 mm [1]. We defined rapidly-enlarging aneurysms as change in z score by 2 or more units on consecutive echocardiograms in patients with baseline medium or large/giant aneurysms.

A literature search was performed with search terms including Kawasaki disease, treatment, cyclophosphamide or Cytoxan using Pubmed and Embase.

This study was approved by the Boston Children’s Hospital institutional review board.

## Results

Among 712 patients with a diagnosis of KD between 1/2006 and 12/2019, 199 (28%) met criteria for CAA on at least one echocardiogram during the first 6 weeks of illness. Of these patients, 10 (5%) received CYC. Seven were male and the median age at diagnosis was 2.0 years (4 months-5 years). Half of the patients had complete KD per AHA criteria. Table 1 summarizes demographic and echocardiographic findings. All patients had large and/or rapidly expanding CAAs as the primary indication for CYC. Two patients were febrile at the time of CYC administration (Patients #1 and #2, Table 2). Each had resolution of fever after 1 dose.

**Table 1:** Demographics and echocardiographic findings of patient cohort

Total number of CYC treated patients, n	10
Male sex	70%
Median Age at Diagnosis of KD (range)	2 years (4 months – 5 years)
Race	
• White	• 4
• Asian	• 2
• Hispanic	• 3
• Not available	• 1
Complete KD criteria	50%
Median Day of Illness at 1st IVIG (range)	7 (4–10)
Median Day of Illness at CYC^a^ (range)	22.5 (10–36)
Two doses of CYC	30%
Adjunctive Therapies before CYC	
• Glucocorticoids	• 100%
• Infliximab	• 40%
• Cyclosporine A	• 20%
• Anakinra	• 10%
Median Baseline LAD^b^ Z score (range)	3.8 (0.68–13.4)
Median Baseline RCA^c^ Z score (range)	2.1 (0.1–12.16)
Median LAD Z Max during admission (range)^d^	15.7 (4.7–29.6)
Median RCA Z Max during admission (range) ^d^	11.8 (2.0–21.06)
Number of patients with bilateral aneurysms (n, %)	9 (90%)
Number of patients with giant aneurysms (n, %)	8 (80%)

^a^Cyclophosphamide ^b^Left Anterior Descending Artery; ^c^Right Coronary Artery

^d^Z max: largest measurement of LAD or RCA, at any point of the course

**Table 2:** Patient characteristics, treatment, and duration of follow up

Patient #	1	2	3	4	5	6	7	8	9	10
**Sex**	M	F	M	M	M	F	M	M	M	F
**Age at Dx, yrs**	2.1	3.6	1.5	5.2	5.5	2.4	0.3	3.3	2.3	0.9
**# of KD criteria**	5	3	2	4	5	4	2	3	4	3
**DOI at 1st IVIG tx**	4	4	5	8	6	10	6	9	6	8
**# of IVIG doses**	3	2	3	3	2	2	2	2	2	2
**# of IVMP pulses**	2	3	1	3	3	0	0	0	0	0
**DOI at start of main-tenance IVMP 2 mg/kg/day**	23	31	20	16	27	10	6	9	6	8
**Other immunomodulatory treatments**	IFX ×2	IFX	IFX, CSA	CSA	IFX	Anakinra	None	None	None	None
**DOI at CYC**	23 and 32	34	20	25 and 37	31 and 38	25	21	13	10	16
**# of CYC doses**	2	1	1	2	2	1	1	1	1	1
**Duration since dx at last follow up**	11y 10mo	8 yr 11mo	0.5mo	3 yr 10mo	2 yr 11mo	3 yr 1mo	9mo	1 yr 10mo	3mo	2mo
**Initial LAD z score**	0.68	NL	6.08	4.66	1.77	13.06	2.96	10.7	13.4	1.85
**Initial RCA z score**	NL	NL	1.22	3.05	0.1	5.99	2.92	n/a	2.0	4.0
**Max z score at CYC tx, LAD**	27.2	29.57	31.27	17.3	19.9	20.35	5.39	10.12	15.5	4.7
**Max z score at CYC tx, RCA**	22.3	10.5	13.6	13	14.2	7.4	6.67	15.2	2.0	9.8
**Max Z score at last follow up, LAD**	13.5	1.13	31.1	0.56	5.0	13.9	1.02	1.16	7.7	0.4
**Max Z score at last follow up, RCA**	28.5	0.12	13.5	7.32	6.0	1.1	1.78	19.0	1.3	4.1

*Yrs* years, *tx* treatment, *DOI* day of illness, *IVIG* intravenous immunoglobulin, *IVMP* intravenous methylprednisolone, *IFX* infliximab, *CSA* cyclosporine A, *CYC* cyclophosphamide, *LAD* left anterior descending (proximal), *RCA* right coronary artery, *CA* coronary artery dimensions; Normal^α^: z score ≤ 2; CAA^β^: coronary artery aneurysms, z > 2.5

**Fig. 1:**
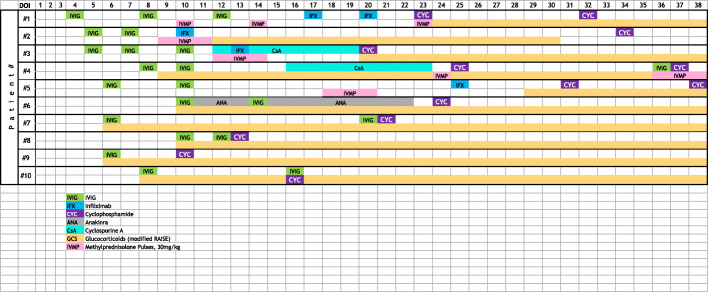
Timeline of medication administration per Day of Illness (DOI)

All patients were treated with IVIG 2 g/kg between days 4–10 of illness and received medium- or high-dose aspirin (ASA) as per AHA guidelines (Table 2) [1]. Half of the patients (5/10) received primary intensification with corticosteroids due to CAA on initial echocardiogram with a modified RAISE (Randomized controlled trial to Assess Immunoglobulin plus Steroid Efficacy) regimen of intravenous methylprednisolone (IVMP) 2 mg/kg/day divided BID with transition to oral prednisolone [12]. All but one patient were re-treated with IVIG. Five patients received IVMP pulses (30 mg/kg, maximum dose of 1 g) in addition to modified RAISE (Table 2**,** Fig. 1). Other immunomodulatory agents used before CYC included: infliximab (*n* = 4), cyclosporine (*n* = 2), and anakinra (*n* = 1). All patients received antithrombotic therapy per AHA guidelines [1], including low dose ASA, clopidogrel, enoxaparin, and/or warfarin. One patient was also treated with abciximab.

Seven patients had CAA at diagnosis (Table 2). Two patients (Patients #1, #2) were diagnosed on day of illness (DOI) 4 and had normal baseline echocardiograms. CAA were subsequently visualized on days 16 and 23, respectively. Patient #7, was diagnosed on DOI 6 and CAA were seen on day 16. All patients had involvement of right and left coronary systems, except for Patient #9, who had a rapidly enlarging aneurysm of the LAD (defined as increase in z score by 2 or more), mild dilatation of the circumflex, and an unusual conical appearance of the proximal RCA. At the time of the first CYC dose, eight patients had at least one z score > 10; six patients had z scores > 10 for both LAD and RCA (Table 2).

The median time to first CYC dose was 22.5 days (10–36 days) from fever onset. There was a trend towards administration of CYC earlier in illness in the more recent time period. Specifically, the median time for the first CYC dose in the 5 patients treated between 2008 and 2016 was 25 days compared to 16 days for patients treated between 2016 and 2019. The coronary artery dimensions in seven patients stabilized after a single CYC dose. Three patients received a second dose of CYC 7–12 days after first due to progressive coronary enlargement; all were in 2008–2016 cohort. One patient’s CYC dose was decreased to 5 mg/kg due to concern for increased toxicity after prior cyclosporine administration. One patient’s dose was calculated per ideal body weight given obesity. Other than continuing oral steroids, no further immunosuppressive treatment was administered after CYC in any patient.

**Fig. 2:**
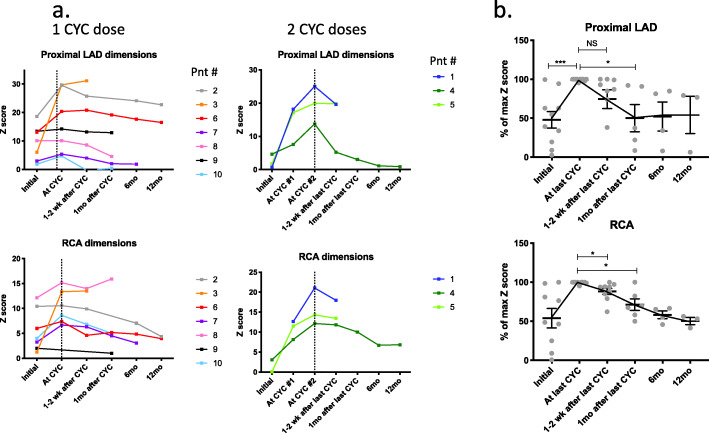
**a** Coronary dimensions over time: absolute z score values. **b** Normalized z scores of LAD and RCA dimensions. The data expressed as a percentage of highest Z score value per patient with highest equal 100%. Each dot represents individual patient. NS not significant, *p < 0.05, **p < 0.005, ***p < 0.0005

Follow-up echocardiography revealed stabilization and/or improvement in CA size following the last dose of CYC (Fig. 2). At most recent follow-up, median Z score was 3.08 for the proximal LAD and 5.05 for the RCA. Patient #7 had normalization of coronary artery internal lumen diameter noted 9 months after diagnosis. Patient #2 had z scores < 2.5 of the proximal RCA and LAD on the last follow-up at over 8 years after KD diagnosis but was noted to have a persistent, distal LAD aneurysm of 4 mm with obstructing thrombus. Angiogram revealed robust distal flow and the patient had a normal stress echocardiogram. Four patients had improved CAA with z score < 10, and 4 patients had persistent large/giant CAA on last follow up. In regards to the two patients who had larger z scores of the RCA in later follow up, the coronary artery enlargement occurred after the window of active inflammation. Furthermore, as the RCA changes were not accompanied by signs of systemic inflammation, such as fever or elevated inflammatory markers, they were attributed to hemodynamic changes and mechanical stressors on damaged vessel walls. Accordingly, those patients did not receive further immunosuppression.

There were no major adverse cardiac events in this cohort over a median follow-up of 2.3 years (range 2 weeks-11.8 years). CYC was generally well tolerated. No infections or bladder toxicity occurred. Significant neutropenia developed in one child with a preexisting history of neutropenia (absolute neutrophil count 400 at age 2 months). He had normal ANCs both at admission for KD (ANC = 5900) and prior to CYC treatment (ANC = 2200). Before administering CYC, hematology was consulted and agreed with the treatment plan. On day 9 after one dose of CYC, his ANC was 100. He had no infectious complications and his neutrophils improved without intervention at 3 months follow-up (ANC = 1270). The other 9 cases had no evidence of bone marrow suppression. There were no discernible long-term side effects from the CYC during the available follow up period.

## Discussion

We report the largest series to date of KD patients treated with CYC for large or rapidly progressive CAA. Following treatment with 1–2 doses of CYC, coronary artery dimensions stabilized, and no further immunosuppressive treatment was prescribed other than a tapering course of prednisone. Importantly, CYC was generally well tolerated, with no serious infections or long-term toxicities.

Similar to the pathogenesis of some other vasculidites, monocytes/macrophages, T-lymphocytes and IgA plasma cells play a major role in the formation and maintenance of inflammatory infiltrates in the arteries in KD [13–15]. A recent study by Noval Rivas et al. has strengthened the evidence for potential role of secretory IgA deposition in coronaries of KD patients [16]. We hypothesize that in progressive KD, these active immune cells/complexes are not fully suppressed by steroids or selective anti-cytokine therapies, and hence require direct cytotoxic alkylating mechanism of cyclophosphamide, which has been effectively used in treatment of other IgA mediated vasculidities [17, 18]. We compared clinical management and outcomes in our series to those of 7 previously reported cases in 3 published series [9–11]. CYC-treated KD cases varied significantly with respect to indications, dosing, duration and use of other medications **(**Table 3).

**Table 3:** Summary of Published Series of Patients with Severe Kawasaki Disease Treated with Cyclophosphamide

Article	# of pnts	Age at Dx	Sex	CYC dose	Route	CYC duration	Prior Tx	Follow up	Outcome
**Wallace CA et al, 2000** [9]	2	0.9 yr, 2.7 yrs.	Male	2 mg/kg/day	IV then PO	1.5-7mo	IVIG, IVMP	2.5 yr and 2.8 yrs.	Normal CAs at last follow up
**Lucron H et al, 2004 **[10]	4	0.3 yr, 0.8 yr, 2 yrs., 4.3 yrs	50% Male	10 mg/kg/day IV, then 2 mg/kg/day PO	IV then PO	IV 2–5 days, PO 6-12mo	IVIG, Plasma-pheresis	8 yrs. and 13 yrs	2/4 deceased, both with myocarditis and CAAs
**Briceno-Medina M et al, 2016** [11]	1	12 yrs.	Male	15 mg/kg/day	IV	4 doses	IVIG, IVMP	3 yrs.	+ CAA, no stenosis or thrombosis
**Current series, 2020**	10	Median 2 yrs	50% Male	10 mg/kg/dose	IV	1–2 doses	IVIG, IVMP, IFX, CsA, ANA	Median 2 yrs. 4mo	Mean (median) z scores at last follow up: LAD 7.5 (5.0), RCA 8.3 (6.0)

*Pnts* patients, *Dx* diagnosis, *CYC* cyclophosphamide, *IV* intravenous, *PO* oral, *IVIG* intravenous immunoglobulin, *IVMP* intravenous methylprednisolone, *CAs* coronary arteries, *NL* normal, *CAA* coronary artery aneurysms, z > 2.5, *IFX* infliximab, *ANA* anakinra, *CsA* cyclosporine A, *LAD* left anterior descending (proximal), *RCA* right coronary artery

In our cohort, we used CYC for worsening CAA, whereas prior case reports described use of CYC for refractory fever and/or persistent KD criteria. Wallace et al. [9] described two KD patients who were treated with IV CYC (2 mg/kg/day) for recurrent clinical symptoms and a rise in C-reactive protein after discontinuation of IVMP. Oral CYC and prednisone were continued for 1.5 and 7 months. One of these patients had a medium sized aneurysm (5.5 mm) that remodeled to normal dimension by 2.5 years, and the other never developed aneurysms. A retrospective cohort of 52 KD patients treated from 1984 to 2003 in France described four patients treated with CYC [10]. They received CYC (10 mg/kg IV × 2–5 days, +/− oral for 6–12 months) due to persistent fever after multiple doses of IVIG. No patient in this cohort was on concurrent daily corticosteroids; one patient received one dose of IVMP. Two patients survived with 8 and 13 years follow up, whereas the two patients who received 5 consecutive days of CYC died on Days 15 and 64. Briceno-Medina et al. described a case of a 12-year-old boy with KD complicated by multiple saccular aneurysms of the left main coronary artery, LAD, circumflex and RCA as well as a multiple non-coronary arterial aneurysms [11]. He was treated with standard KD therapy plus methylprednisolone and CYC 15 mg/kg/day × 4 doses. At 3 years of follow up, he had no evidence of coronary artery stenosis or thrombus, although giant CAAs persisted in both the RCA and LAD.

Our regimen of 1–2 doses of CYC differs from previous reports of prolonged treatment in refractory KD. A standardized dosing regimen of 10 mg/kg/dose IV was utilized, based on the use of CYC in other vasculitic and rheumatologic diseases [19]. The more recent patients in our cohort received CYC earlier in their course, reflecting an increased institutional comfort with use of CYC in KD patients with severe coronary artery involvement.

Aside from self-resolving neutropenia in one patient as detailed above, there were no other reported short-term adverse events, consistent with reported experience [9–11]. Long-term toxicity of CYC in regard to secondary malignancies and infertility was not reported, and would not be expected given the young age of the patients and the low cumulative dose of CYC.

Limitations of this study are inherent to retrospective data collection, including lack of standardized initial treatment, varying timing of CYC, and use of other adjunctive anti-inflammatory therapies. Indeed, regimens in our cohort reflect secular trends of treatment in KD. While we saw stabilization and/or improvement in CAA in all patients after CYC therapy, it is not possible to ascribe causation of CAA improvement given the observational nature of our study and we cannot exclude the impact of treatments prior to CYC initiation. While all patients received steroids and multiple other anti-inflammatory therapies that may also have influenced CAA outcome, it is notable that 8/10 patients received CYC due to continued CAA enlargement despite other therapies, suggesting that CYC had a distinct, beneficial anti-inflammatory effect. The natural history of CAA in KD is often stabilization of CAA size dimensions in the 3rd to 6th weeks of illness; in some patients, CYC was administered in this timeframe. However, three patients were treated with CYC before Day 17, a time period in which coronary dimensions often continue to expand, and all three experienced rapid improvement in their z scores after CYC. It should also be noted that all patients continued on corticosteroids following CYC administration, per RAISE protocol routinely followed in KD patients with aneurysms at our institution.

Regarding potential other considerations for CYC use in KD treatment, the cost of CYC is significantly less as compared to IVIG and biologics, which may be an important consideration in less resourced clinical settings, although the higher risk of infection should be carefully considered in such cases.

## Conclusion

This series describes CYC administration in patients with KD who had rapidly progressive or large CAA despite multiple anti-inflammatory treatments. In this setting, CYC administration was associated with stabilization of CAA dimensions and a favorable safety profile.

## Data Availability

The datasets generated during the current study are not publicly available due to its link to patient identifying information but are available from the corresponding author without patient identifiers on reasonable request.

## Abbreviations

KD: Kawasaki disease
CAA: Coronary artery aneurysms
CYC: Cyclophosphamide
IVIG: Intravenous immunoglobulin
AHA: American Heart Association
LAD: Proximal left anterior descending coronary artery
RCA: Right coronary artery
IRB: Institutional Review Board
ASA: Aspirin
RAISE study: **R**andomized controlled trial to **A**ssess **I**mmunoglobulin plus **S**teroid **E**fficacy for Kawasaki disease
IVMP: Intravenous methylprednisolone
DOI: Day of illness
ANC: Absolute neutrophil count
